# Plant pathogen effector proteins as manipulators of host microbiomes?

**DOI:** 10.1111/mpp.12628

**Published:** 2018-01-25

**Authors:** Nick C. Snelders, Graeme J. Kettles, Jason J. Rudd, Bart P. H. J. Thomma

**Affiliations:** ^1^ Laboratory of Phytopathology Wageningen University and Research 6708 PB Wageningen the Netherlands; ^2^ Biointeractions & Crop Protection Rothamsted Research Harpenden Hertfordshire AL5 2JQ UK

To understand the mechanisms underlying disease development in plants, molecular plant pathology research has mostly focused on the characterization of direct interactions between plant pathogens and their hosts. Collectively, this research has demonstrated that plants sense microbial invaders using various types of receptors (recently coined as ‘invasion pattern receptors’, IPRs) that sense microbial invasion and activate defence responses upon recognition of various molecular patterns that betray microbial invasion (recently coined as ‘invasion patterns’, IPs) (Cook *et al*., 2015). While these IPRs comprise cell surface‐localized as well as intracellular receptors, IPs comprise microbe‐associated molecular patterns (MAMPs) and other microbially secreted components, as well as host‐derived damage‐associated molecular patterns (DAMPs) (Cook *et al*., 2015).

In order to successfully colonize their hosts and subvert immune responses, plant pathogens secrete molecules, so‐called effectors, during attempted host ingress (Cook *et al*., 2015; Rovenich *et al*., 2014). According to the initial, narrowest, definitions, effectors are small, cysteine‐rich proteins that function through the manipulation of plant immune responses. However, ongoing research has revealed that effectors may have other functions as well, such as roles in pathogen self‐defence or liberation of nutrients from host tissues (Fatima and Senthil‐Kumar, 2015; Rovenich *et al*., 2014). Moreover, it is generally appreciated that other types of microbially secreted molecules, such as secondary metabolites and small RNAs (Wang *et al*., 2016), may exert prototypical effector functions. Furthermore, it is accepted that effectors are not exclusively secreted by pathogens, as homologous molecules are employed by other types of symbiotic organisms, such as endophytes and mutualists, and even by saprophytes (Rovenich *et al*., 2014). Consequently, more recently, it has been proposed that rather than being small, cysteine‐rich proteins that function through the manipulation of plant immune responses, effectors should be defined as microbially secreted molecules that contribute to niche colonization (Rovenich *et al*., 2014).

Similar to other higher organisms, plants associate with a plethora of microbes that collectively form its microbiome. The phyllosphere comprises all aerial parts of the plant and is commonly colonized by diverse microbial communities (Vorholt, 2012). However, the most extensive microbial host colonization occurs below ground. The soil is a hotspot of microbial life, as microbial communities generally display great diversity and reach high densities. In particular, the narrow zone in close proximity to the roots, also known as the rhizosphere, is extremely microbe rich as it attracts microbes from the surrounding soil and allows them to thrive on plant‐derived root exudates (Bais *et al*., 2006). Over recent years, the plant microbiome has gained increasing attention. Metagenomic studies have greatly enriched our knowledge of the composition of plant microbiomes and have led to its recognition as a key factor for plant health (Berendsen *et al*., 2012). The role of the rhizosphere microbiome in disease suppression has been particularly well described. It is currently generally appreciated that plants exploit root exudates to increase microbial activity on pathogen attack, and specifically attract beneficial microbes from the very diverse microbial community residing in the bulk soil (Berendsen *et al*., 2012). Consequently, plants select microbial communities around their roots that function as an additional layer of defence. One of the best‐studied examples is the reduced incidence and severity of take‐all disease caused by the fungus *Gaeumannomyces graminis* var. *tritici* which typically follows a severe disease outbreak in a monoculture of wheat or barley. This phenomenon is known as the so‐called ‘take‐all decline’ and is associated with the elevated presence of antagonistic *Pseudomonas* spp. that suppress the soil‐borne fungal pathogen.

Like all microbes, plant pathogens are under strong selective pressure exerted by co‐inhabiting microorganisms. These microbiota members influence each other, both positively and negatively, through secreted molecules. A significant part of these molecules function through their antimicrobial activity and involve hydrolytic enzymes, antibiotics, toxins and volatiles (Compant *et al*., 2005). In addition, microbes strongly compete with each other for nutrients and essential elements. Importantly, these processes often involve secreted molecules. Siderophores and haemophores are well‐studied molecules secreted by plants and soil microbes to scavenge metal ions and facilitate their uptake (Compant *et al*., 2005). Obviously, the above‐mentioned antibiosis and competition for nutrients also impact microbial plant pathogens, and represent two important factors in disease suppression. However, other mechanisms responsible for disease suppression have also been reported. For example, beneficial rhizobacteria indirectly affect pathogens through the induction of systemic resistance in plants. Interestingly, rhizobacteria do so through various mechanisms, including the secretion of particular volatiles, antibiotics and siderophores that prime the plant's immune system for pathogen attack (Compant *et al*., 2005).

To date, the study of the plant microbiome and biocontrol has exclusively investigated the influence of microbial communities on plant pathogens and host defence activation. However, the manipulation of these communities by plant pathogens in return, during host colonization to promote this process, as well as during free‐living life stages outside the host, remains unexplored. Arguably, effector proteins may act as exquisite tools for the interaction with other microbes. This hypothesis may be supported by observations that, across numerous pathosystems, and despite significant effort, the functions of many effectors in terms of host plant manipulation remain unknown. Although this may derive from overlapping effector functionalities with plant targets, it may also be that some secreted protein effectors might instead be targeting the local microbial community. In addition to during host colonization, microbiota‐manipulating effectors may also be important for saprophytic survival during free‐living life stages outside the host. Arguably, non‐pathogenic saprophytes may employ similar molecules to sustain themselves in the presence of other microbes, whereas endophytes and mutualists can be anticipated to secrete similar effectors to outcompete other microbes in the process of host colonization. With this in mind, effector proteins in general could be broadly classified into three groups: (1) plant‐targeting effectors; (2) multifunctional effectors targeting plants and microbes; and (3) microbe‐targeting effectors.

- Group (1) effectors have a role solely in the manipulation of the host organism. This includes pathogen proteins which suppress pathogen‐associated molecular pattern‐triggered immunity (PTI) and may be recognized in a gene‐for‐gene manner to induce either host resistance or susceptibility. It also includes effectors demonstrated to have multiple roles in host manipulation, such as the SnTox1 effector from *Parastagonospora nodorum* (Liu *et al*., 2016).
- Group (2) effectors have roles in the manipulation of both the host and the local microbial community. Such a group is probably dominated by proteins with broad‐spectrum activity targeting highly conserved physiological processes functional in both plants and microbes. For instance, effector proteins involved in self‐defence towards antimicrobial components, such as hydrolytic enzymes secreted by plant hosts, can also be expected to offer protection against similar components secreted by competing microbes. In addition, plant pathogens can also be anticipated to secrete effector proteins with simultaneous phytotoxic and antimicrobial activity that affect both host and other microbes, such as the recently described Zt6 effector from the wheat pathogen *Zymoseptoria tritici* (Kettles *et al*., 2017).
- Group (3) effectors are highly specialized to target or disrupt processes specific to microbes, and thus may have distinct biochemical properties from those designed to target analogous mechanisms in plants. This group may also include those which act in an indirect fashion, for example by establishing local nutrient deprivation, or by affecting communication between plants and beneficial microbes. Finally, pathogens may secrete effectors to recruit cooperative microbes that offer protection against microbial competitors, or that aid in host colonization. This group of effectors may also play important roles for endophytic and saprophytic species.

Given their potential diversity, the task of identifying effector proteins involved in microbiota manipulation may appear to be daunting. However, expression profiling of genes coding for secreted proteins and direct identification by proteomics approaches of candidates during host colonization have proven to be successful for the identification of host‐manipulating (group 1) effectors. Arguably, microbiota‐manipulating effectors (groups 2 and 3) require different transcriptional triggers compared with the canonical effectors characterized to date, and probably display different transcriptional patterns compared with effectors dedicated purely to host manipulation. For example, elevated expression following complete colonization of the host may be unusual for host‐manipulating (group 1) effectors, but may be commonplace for effectors intended to limit nutrient scavenging by competing microbes. Furthermore, distinct transcriptional signatures probably exist within each pathogen which may be highly dependent on pathogen lifestyle and exposure to microbial antagonists within a particular niche. Thus, transcriptomic approaches can be exploited to monitor the induction of effector genes under *in vitro* conditions that mimic microbial encounter. However, great care must be taken in the interpretation of these experiments.

It is possible that effector proteins that are relevant for survival in microbial communities are shared between closely related species that operate in the same niche. Hence, comparative genomics between saprophytic and pathogenic relatives can be used to identify core effector gene catalogues. For the effector categories introduced above, host‐manipulating group 1 effectors probably belong to a single or small group of pathogen(s) and play highly specialized roles in the manipulation of perhaps a single (or small number of) host(s). Host‐ and microbe‐targeting group 2 effectors probably exhibit a broader distribution not only amongst plant pathogens, but also amongst non‐pathogenic species, because of their ability to influence microbe–microbe interactions. Finally, microbe‐targeting group 3 effectors probably display the broadest distribution of all, encompassing plant pathogens, endophytes and saprophytes. To this end, transcriptomic analyses and comparative genomics approaches complement each other and can be used in parallel to identify relevant effector candidates. Subsequently, functional screens aimed to determine their direct effect on other microbes should reveal whether or not the effector candidates have potential microbiota‐manipulating abilities. An initial (and potentially overlooked) medium‐ to high‐throughput screen might be to first test whether candidate proteins can be expressed in either prokaryotic or eukaryotic recombinant expression systems. Our recent discovery of the multifunctional Zt6 effector from *Z. tritici* initially came from our inability to express full‐length recombinant protein in either *Escherichia coli* or *Pichia pastoris* expression systems, potentially due to toxicity (Kettles *et al*., 2017). This contrasts with most other tested *Z. tritici* candidate effectors, which express relatively well in either system, albeit often to different levels. The availability of specialist *E. coli* expression strains (e.g. SHuffle, New England Biolabs, Ipswich, MA, USA), designed to express cysteine‐rich eukaryotic proteins with minimal inclusion body formation, makes this toxicity screen viable. Although this type of screen based on negative results is probably not optimal, it could be used as a baseline for then testing the relative ability to generate subtle mutant versions of these proteins for more direct testing, or to test for toxicity responses via other transient expression systems, perhaps even using agroinfiltration as a route to determine whether the toxicity is broad or selective. It may even be possible to recover protein from such a system for direct testing on microbes. In addition, screens can be aimed at the identification of effector candidates involved in the recruitment of cooperative microbes by determining their ability to promote the growth of other microbial species. Finally, irrespective of observations during initial screens, gene functional analysis will be required to validate the relevance of the effectors in the biological context and to confirm their role in microbial interactions.

Unveiling the roles of plant pathogen effector proteins in the manipulation of microbiota will add to our fundamental understanding of the mechanisms contributing to disease establishment, and could potentially lead to improved disease control methods. Current crop disease control is heavily reliant on the application of synthetic fungicides and bacteriocides. However, pathogen resistance to chemical control has, in some cases, become widespread. In addition, soil‐borne pathogens are especially difficult to control because of their persistent resting structures. Therefore, the biocontrol of plant pathogens using antagonistic microbes is an alternative option. Nevertheless, the biocontrol of pathogens is not always consistent and could be improved to become a more reliable disease control method. To this end, the characterization of microbiota‐manipulating effectors can contribute to more targeted biocontrol strategies, as it allows for the selection of antagonistic microbes that are insensitive to, or can interfere with, the activities of pathogen effectors. In addition, similar to previously identified effector proteins, plants may have evolved IPRs to recognize microbiota‐manipulating effectors and their activities. Microbiota‐manipulating effectors thus represent an interesting pool of unexplored avirulence factors for which recognition in particular plant genotypes may exist and may help to identify or engineer novel immune receptors that may contribute to improved pathogen resistance in crops. Finally, given that many microbes secrete antimicrobial molecules, but are themselves immune, what are the mechanisms of self‐protection? An understanding of these fundamental aspects of microbe–microbe interactions on plants may provide a future source of targets for intervention and disease control.
